# Virologic and Immunologic Characteristics in Mature Ducks with Acute Duck Hepatitis A Virus 1 Infection

**DOI:** 10.3389/fimmu.2017.01574

**Published:** 2017-11-16

**Authors:** Sai Mao, Mingshu Wang, Xumin Ou, Di Sun, Anchun Cheng, Dekang Zhu, Shun Chen, Renyong Jia, Mafeng Liu, Kunfeng Sun, Qiao Yang, Ying Wu, Xinxin Zhao, Xiaoyue Chen

**Affiliations:** ^1^Institute of Preventive Veterinary Medicine, Sichuan Agricultural University, Chengdu, China; ^2^Key Laboratory of Animal Disease and Human Health of Sichuan Province, Sichuan Agricultural University, Chengdu, China; ^3^Avian Disease Research Center, College of Veterinary Medicine, Sichuan Agricultural University, Chengdu, China

**Keywords:** duck hepatitis A virus 1, hepatitis model, course of viremia, antibody responses, innate immune responses, virus clearance

## Abstract

Duck hepatitis A virus 1 (DHAV-1) infection in mature ducks has previously been proposed as a small-animal model for human hepatitis A. However, basic research on the outcome of DHAV-1 infection in mature ducks is limited. Here, we examined the course of viremia, the characteristics of antibody responses, and the profiles of plasma cytokines in mature ducks infected with DHAV-1. During the course of infection, the viremia was detectable soon after infection and persisted for 196 days, however, the ducks presented as clinically asymptomatic. Specific and timely immunoglobulin G (IgG), IgM, and IgA1 responses were elicited. At the same time, extensive inhibition of viral replication was observed with increasing IgG concentration. With respect to pattern-recognition receptors, *TLR-7* was mainly involved in triggering the innate defense against the DHAV-1 infection. In addition, plasma immune analytes were measured and were determined to have bidirectional roles in virus clearance. It was concluded that DHAV-1 spreads quickly in blood. The spontaneous clearance of DHAV-1 during asymptomatic infection in mature ducks depends on the cooperation of timely antibody responses and alert innate immune responses. Moreover, the delayed clearance may be associated with a weak interferon-γ-producing CD8+ T cell response. This study allows us to reveal the mechanism of clearance and persistence of DHAV-1 infection in mature ducks. We anticipate that it will provide a basis for future studies focused on defining the nature mechanisms involved in the clearance and persistence of human hepatitis virus.

## Introduction

Duck hepatitis A virus 1 (DHAV-1), a single-stranded positive-sense RNA virus categorized in the genus *Avihepatovirus* in the family *Picornaviridae*, was first reported in Long Island, New York, by Levine and Hofstad in 1945 and it subsequently spread around the world (1, 2). It is the causative agent of Duck Viral Hepatitis in both young ducklings and adult ducks. Pathogenicity studies of DHAV-1 have customarily focused mainly on young ducks, which usually manifest an acute infection and high mortality with depression and feather disorders observed in the clinic, and hemorrhages and liver necrosis revealed through pathology (3, 4). Although the host innate immune responses in the development of acute hepatitis in DHAV-1-infected ducklings had been investigated adequately (3, 5, 6), corresponding research on adult ducks is rare (7). Recently, however, based on pathological research, mature ducks infected with DHAV-1 have been proven as small-animal models for human hepatitis A (8). Thus, advanced research on virus–host relationships in DHAV-1 infection in mature ducks is especially valuable, which will be beneficial in illuminating the development of human hepatitis.

The innate immune response is the first line of host defense, initiated by the rapid recognition of viral components by pattern-recognition receptors (PRRs), such as toll-like receptor (TLR) 7 and 3, retinoic acid inducible gene 1 (RIG-1), and melanoma differentiation factor 5 (MDA5), all of which are usually involved in combatting RNA virus infection (9–12). All four PRRs are involved in DHAV-1 infection in young ducklings in the acute infection phase (3). The activation of the innate immune system induces an antiviral state by producing type I interferons (IFNs) and pro-inflammatory cytokines, which are critical mediators of antiviral defenses (13, 14). In DHAV-1-infected ducklings, type I and II IFNs and certain interleukins (ILs) such as IL-2 and IL-6 are significantly induced (3, 5, 6). However, the expression of cytokines such as IFN-α and IL-6 could be influenced by the age of the ducks, similar to the situation observed for the above-mentioned PRR expression (3). Moreover, most innate immune factors showed tissue expression differences in DHAV-1 infection ducks (7). Therefore, it is necessary to determine the expression profiles of innate immune factors and their relationships with DHAV-1 infection in mature ducks.

Adaptive immunity is crucial in protecting the host from infection and in eliminating pathogens. A strong IFN-γ-producing CD8+ T cell response is associated with viral clearance (15). Serum antibodies are also routinely tested as part of the investigation of subjects with suspected liver disease, and a polyclonal increase in serum immunoglobulin levels is frequently seen in patients with liver cirrhosis (16). Moreover, increases in different classes of antibody can be affected by the causative agent and the degree of liver disease (17, 18). To date, studies of antibodies against DHAV-1 have usually been limited to the establishment of detection methods (19, 20) or to the rate of vaccine protection against DHAV-1 in ducklings (21). However, the dynamics of DHAV-1-specific antibodies and their role in DHAV-1 infection have been paid little attention.

In this study, the longitudinal infection course of viremia and the immunological responses in model ducks infected with DHAV-1 were investigated. We analyzed the relationships between the kinetics of viral spread and the induction of humoral and innate immune responses to DHAV-1 in circulation. The results demonstrated that a prolonged, acute infection was developed despite the spontaneous clearance of virus mediated by the immediate induction of immunoglobulin G (IgG) and IL-6 in blood. Moreover, several immunological parameters were identified as likely determinants of the delayed clearance.

## Materials and Methods

### Virus

The DHAV-1 H strain (GenBank: JQ301467.1) was selected to investigate its replication and effects on the immune responses of adult ducks. The virus was propagated in 10-day-old duck embryos by standard procedures and harvested from allantoic fluids of deceased duck embryos at 36–72 h post-infection (4, 22). Virus at a concentration of 4.56 × 10^8^ copies/mL determined by quantitative real-time PCR (qPCR) was used to infect ducks (23). Collected virus was stored sterile at −80°C until use.

### Ducks and Infections

One hundred five 160-day-old female Peking ducks (*Anas platyrhynchos domesticus*), which had been vaccinated with CH60 (DHAV-1-attenuated vaccine) at 1-day-old, were purchased from commercial hatcheries and divided randomly into an experimental group (100 ducks) and a control group (5 ducks). All the ducks were confirmed free of the DHAV-1 pathogen as the viral loads were too low to be detected by the established qPCR technique (23), but were positive for IgG, with a titer of 1:950 detected by an established indirect ELISA (19). The ducks in the experimental group were inoculated by intramuscular injection with 1 mL of allantoic fluid containing 4.56 × 10^8.0^ virus copies. The control ducks were injected with an equal volume of 0.85% physiological saline. The ducks were carefully reared on a free-range farm without history of DHAV-1.

### Samples

Plasma and sera were drawn randomly from five experimental ducks at 0.5, 1, 2, 4, 6, 8, 10, 12, 14, 21, 28, 56, 84, 112, 140, 168, 196, 224, 252, and 280 days post-infection (dpi), respectively. Corresponding specimens were gathered from the control group, termed 0 dpi. In detail, 150 µL of plasma was added into 1 mL of RNAiso Plus (9108, Takara, Japan) as soon as it was pumped from the external jugular vein, and then stored at 4°C pending RNA isolation. Sera were stored sterile at −20°C pending the detection of antibodies.

### RNA Isolation and cDNA Preparation

Total cellular RNA was extracted from 150 µL of plasma using the RNAiso Plus (9108, Takara, Japan) according to the manufacturer’s instructions. An amount of 6 µL of RNA from each specimen was used to synthesize cDNA using the PrimeScript™ RT reagent kit (RR047, Takara, Japan) according to the manufacturer’s protocols, to detect immune-related genes.

### Quantitative Real-time PCR

Viral copies in the isolated RNA were detected according to a previously established one-step real-time Taqman PCR procedure in our laboratory (23). The detection limit of this assay was 166 RNA copies/mL. Expression levels of 17 immune-related genes, *IL-1*β, *IL-2, IL-4, IL-6, IFN-*α, *IFN-*β, *IFN-*γ, *major histocompatibility complex (MHC)-I, MHC-II, C-C motif chemokine ligand (CCL)-19, CCL-21, B cell activating factor (BAFF)*, β*-defensin, TLR-3, TLR-7, RIG-1*, and *MDA5*, and a housekeeping gene (*glyceraldehyde-3-phosphate dehydrogenase, GAPDH*) were determined using the transcriptional cDNA as templates according to previously described protocols (24). The primer sequences for *GAPDH, IL-1*β, *IL-2, IL-6, IFN-*α, *IFN-*β, *IFN-*γ, *MHC-I, MHC-II*, and *TLR-7* have been reported by Adams et al. (25) (Table S1 in Supplementary Material). The primer sequences for *IL-4, CCL-19, CCL-21, BAFF*, β*-defensin, TLR-3, RIG-1*, and *MDA5* have been reported by Ou et al. (24) (Table S1 in Supplementary Material).

### ELISA

The production of specific antibodies (including IgG, IgM, and IgA1) was detected as previously described (19). According to the standard procedures for indirect ELISA, purified viral granules of DHAV-1 were coated overnight at 4°C at the concentrations of 1.79, 2.23, and 2.23 µg/mL for the detection of specific IgG, IgM, and IgA1 in serum, respectively. In addition, serum samples were diluted at 1:80, 1:40, and 1:40 for the detection of IgG, IgM, and IgA1, separately. The diluted samples were incubated at 37°C for 1 h. Goat anti-duck IgG-horseradish peroxidase (HRP) conjugate (KPL, Gaithersburg, MD, USA), goat anti-duck IgM-HRP conjugate (ABIN568547, Antibodies-online), and mouse anti-duck IgA1 (Abd Serotec, Kidlington, UK) labeled using a Lightning-Link™ HRP Conjugation Kit (Innova Biosciences, Cambridge, UK) were used at a concentration of 1:300, 1:1,800, and 1:800, respectively. The optical density (OD) value was measured at double wavelengths of 450 and 630 nm (OD_450_–OD_630_) with a micro-plate spectrophotometer (Model 680, Bio-Rad). The antibody titers were calculated using the established equations, *y* = 1.363 + 1.954 *x* (*r*^2^ = 0.983), *y* = 1.141 + 2.228 *x* (*r*^2^ = 0.970), and *y* = 1.103 + 1.559 *x* (*r*^2^ = 0.995) for IgG, IgM, and IgA1, respectively, on the basis of each OD value.

### Statistical Analyses

Relative expression data for immune-related genes were analyzed using the 2^−ΔΔCt^ method (26). Figures were generated by GraphPad Prism 6 software. Correlation of immune-related genes, virus, and antibodies was determined using Pearson correlation analysis by SPSS software (IBM, Armonk, NY, USA) (27). These correlated factors were visualized as a regulatory network by Cytoscape software (28).

## Results

### Course of Acute DHAV-1 Infection in Mature Ducks

**Figure 1:**
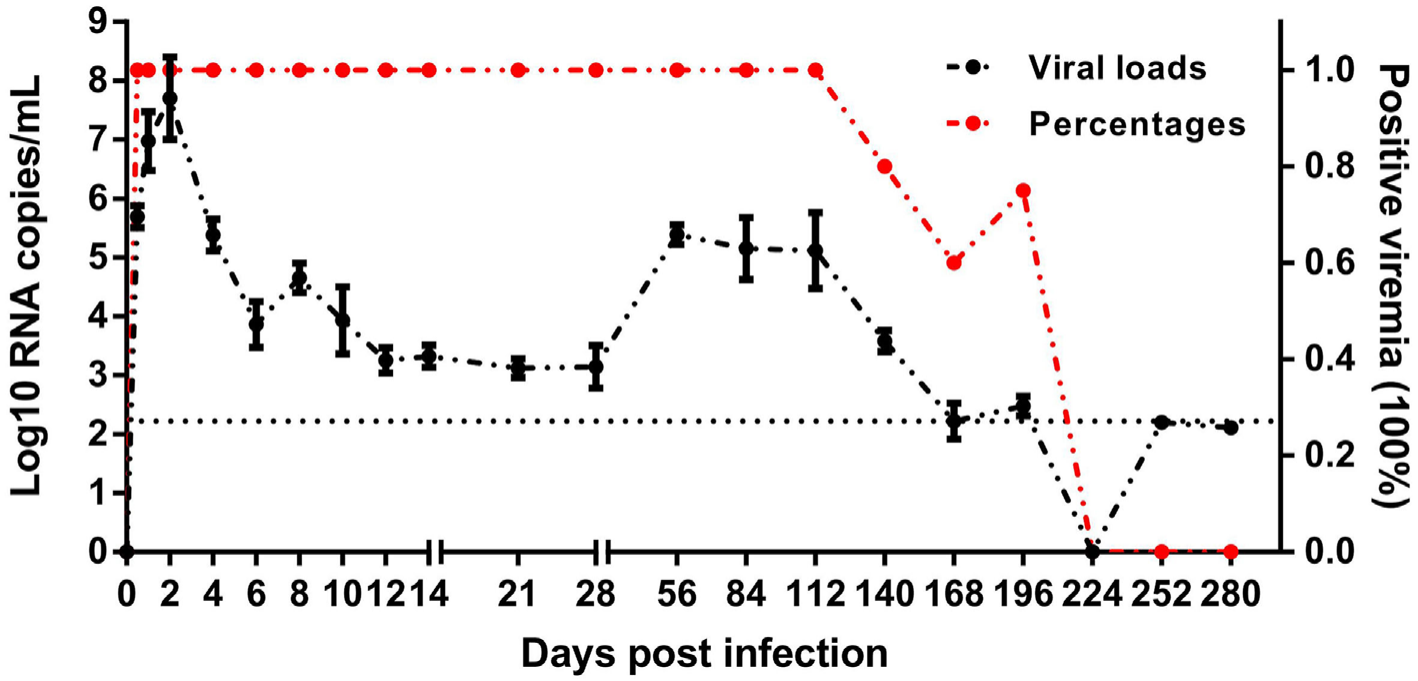
Course of duck hepatitis A virus 1 viremia (Log10 viral RNA copies/mL) in mature ducks after acute infection. The control group is shown as 0 dpi. Percentages represent the number of ducks with positive viremia. The number of ducks evaluated at each time point was 5, except for four ducks at 196 dpi. The dotted line represents the low limit of detection.

**Figure 2:**
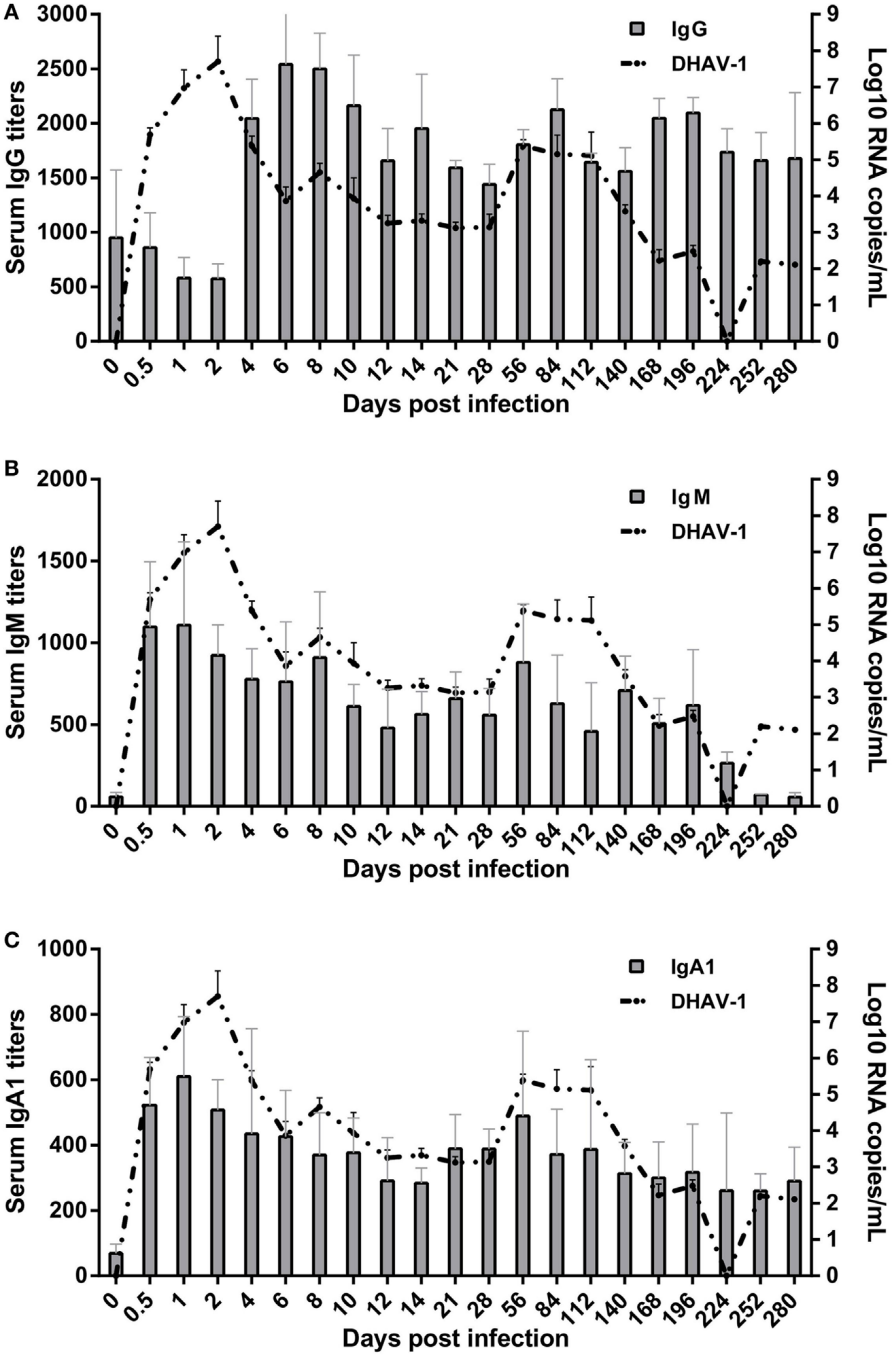
Kinetics of specific antibody interactions with duck hepatitis A virus 1 in blood. The base levels of immunoglobulin G (IgG) **(A)**, IgM **(B)**, and IgA1 **(C)** were 1:126, 1:60, and 1:40, respectively, which were calculated on the basis of their individual cutoff points (19). The antibody titers at 0 dpi represent the residual levels induced by previous immunization with titers of 1:950 ± 620, 1:57 ± 29, and 1:69 ± 28 for IgG, IgM, and IgA1, respectively.

The mature ducks in this study were asymptomatic without significantly elevated serum bilirubin, but with elevations of serum alanine aminotransferase (ALT) activity as described previously (8). This permitted us to study features of asymptomatic hepatitis. Serial viral load monitoring of DHAV-1 RNA in the blood showed these ducks were viremic as soon as 0.5 days after exposure, and viral load increased very rapidly, reaching a peak titer of 10^7.7^ RNA copies/mL by day 2 (Figure 1), preceding significant increases in serum IgG anti-DHAV-1 (Figure 2A). Afterward, the viral titer was rapidly decreased by >10^3^-fold by day 10 with little rise in serum ALT, followed by a gentle decline of viral titer with only a 6-fold decrease by day 28 but with a surge in serum ALT activity, probably indicating the non-cytopathic nature of DHAV-1. Subsequently, the virus titer rebounded briefly at 56 dpi and remained relatively stable for the next 2 months. From 140 dpi, the viral copies declined again and finally became undetectable from 224 dpi onward. Hepatitis was first detectable as significantly elevated serum ALT activity at 28 dpi (8).

Indeed, these subjects spontaneously cleared the virus, however, the DHAV-1 RNA remained positive at 196 dpi, more than 6 months after infection, which is considered the common boundary between acute and chronic hepatitis. The delayed elimination of virus in mature ducks showed a competence of DHAV-1 to progress to chronicity, which provide the possibility to study chronic hepatitis.

### Specific IgG, IgM, and IgA1 Antibodies Were Induced Effectively by DHAV-1 Infection in Mature Ducks

Patterns of elevation of serum antibodies were observed to monitor the protection characteristics of humoral immune responses against DHAV-1 infection in mature ducks (Figure 2). The early IgG titer resulting from the preexisting IgG anti-DHAV-1 decreased gradually, accompanied by the replication of virus, until day 2 after inoculation (Figure 2A). At 4 dpi, the remembered IgG response was recalled and evident, which has been reported previously for the activation of secondary humoral immune responses (29). Subsequently, serum levels of IgG anti-DHAV-1 antibody increased rapidly, reaching a maximum titer by day 6, later than the viral peak, and remained remarkably stable for the next 4 days, coinciding with an expectably rapid decrease in viral burden. Another period of stable IgG levels followed, with a relatively lower titer preceding a rebound on day 84 responding to the rebound in viremia at 56 dpi. The IgG level was attenuated slightly toward the end of the infection but was maintained at a relatively high level. Note that there was no surge in IgG when the serum ALT varied. More importantly, the dynamic IgG antibody was inversely correlated with the DHAV-1 RNA load (*P* < 0.01) (Figure 4) in blood.

The specific IgM response was timely and quickly mounted to its peak by 1 day after exposure, preceding the viral peak (Figure 2B). The titer of IgM was remarkably stable for the duration of viremia, falling less than threefold by day 196. Note that it was rebounded briefly at 56 dpi in response to the rebound in viremia. Coinciding with undetectable levels of virus, specific IgM fell gradually toward normal. The kinetics of the IgM response were significantly parallel with the change in viral loads in blood (*P* < 0.05) (Figure 4).

The specific IgA1 response was also positive and rose rapidly, reaching its peak at 1 dpi simultaneous with the IgM peak but with a lower titer and then decreasing gradually to day 14 coincident with the decline of viral burden. Subsequently, in accordance with the rebound of virus at 56 dpi, the titers of IgA1 and IgM rebounded. After which the IgA1 levels declined but peculiarly remained above the baseline for the duration of the study (Figure 2C). However, there was no correlation between specific IgA1 and DHAV-1 (*P* > 0.05), but a significant correlation between IgM and IgA1 (*P* < 0.01) (Figure 4).

Several aspects of the humoral immune responses should be noted. First, three subtypes of antibodies were completely responsive and each soon reached a peak, suggesting that the humoral immune system was not impaired markedly by DHAV-1. Second, the peak times of IgM and IgA1 were before the viral peak time, which was followed by the peak in IgG after which the viral titer soon declined, implying that the IgG antibody was mainly responsible for the clearance of virus. Third, serum ALT did not surge when levels of IgG, IgM, and IgA1 shifted significantly within 10 days (8), suggesting that non-cytolytic antiviral mechanisms were contributed to viral clearance during that period.

### *TLR-7* Played a Vital Role in the Recognition of DHAV-1 Infection in Mature Ducks

**Figure 3:**
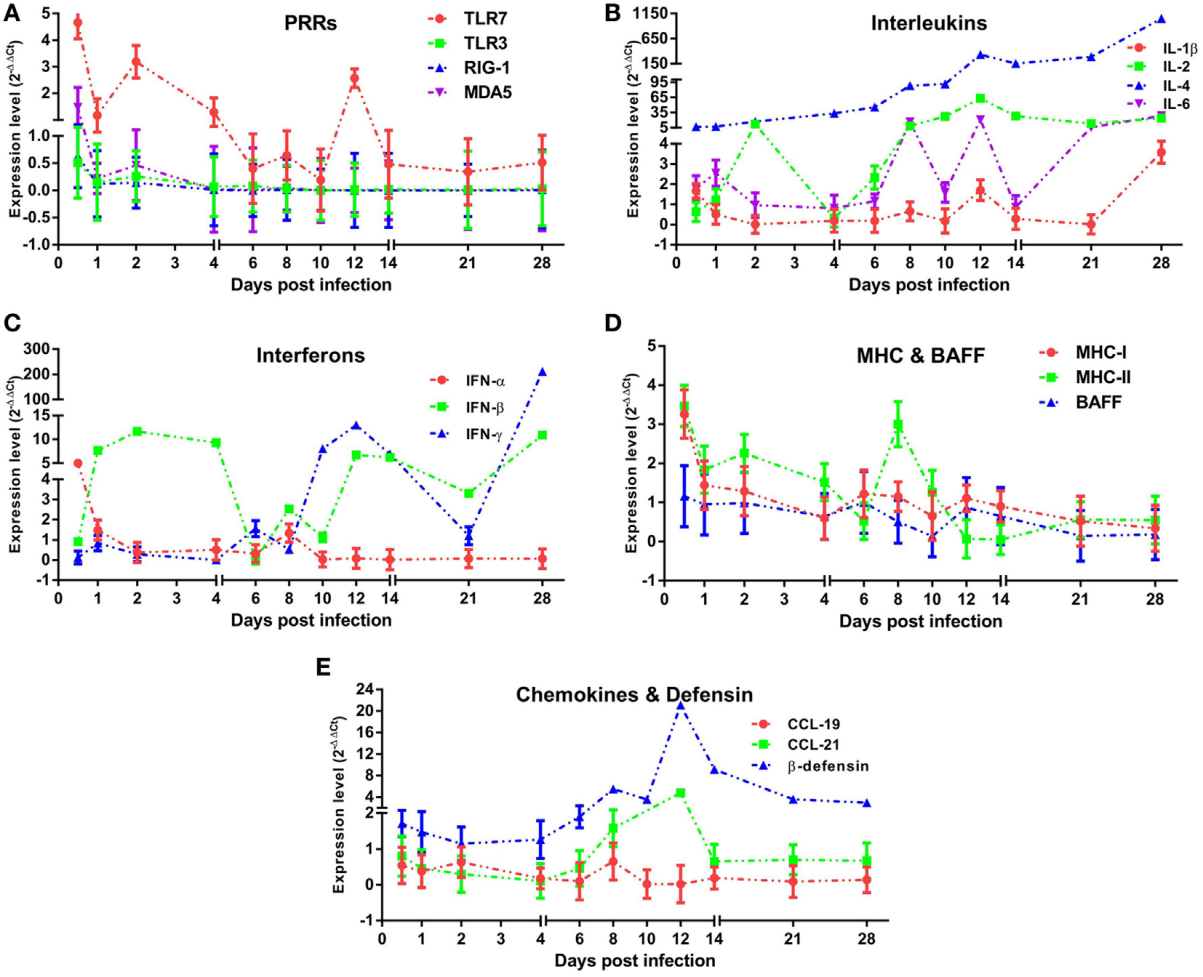
Innate immune responses induced in mature ducks by duck hepatitis A virus 1 infection in the blood. Expression levels of immune-related genes were calculated by the 2^−ΔΔCt^ method using 1 as the baseline. The changes of PRRs **(A)**, Interleukins **(B)**, Interferons **(C)**, MHCs and BAFF **(D)**, and CCLs and β-defensin **(E)** within 1 month post-infection were shown.

**Figure 4:**
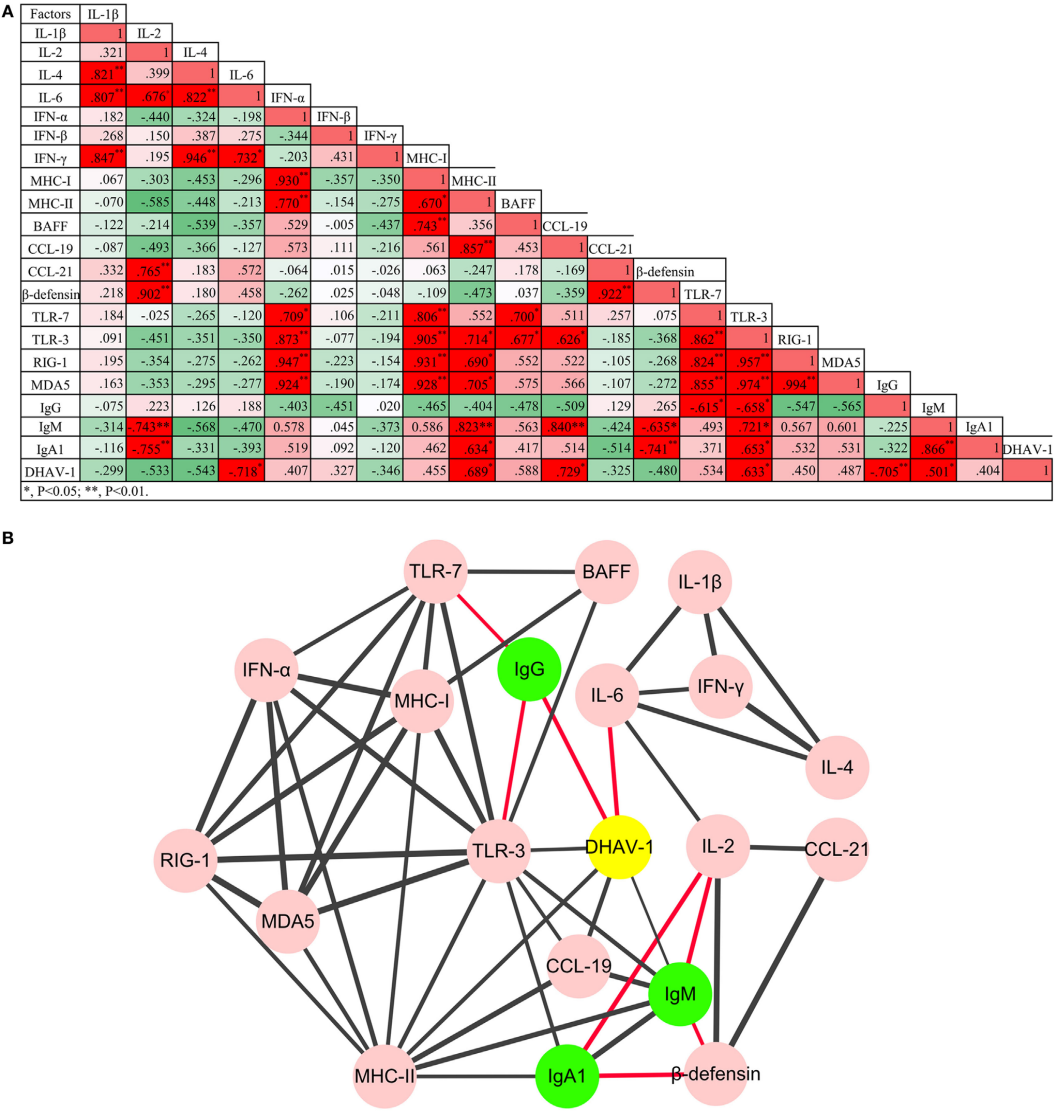
Regulatory networks of innate and humoral immune responses, and the game between duck hepatitis A virus 1 and the host immune system were **(A)** analyzed using Pearson correlation analysis and **(B)** visualized by Cytoscape software (*P* < 0.05 at least). Red lines represent negative correlation, while the black lines represent positive correlation. The thickness of the border represents the intensity of the relationship.

To assess the properties of the innate immune response in asymptomatic DHAV-1 infection in mature ducks, the expression of immune-related genes was measured (Figure 3). In this study, *TLR-7* expression was highly upregulated by day 2 after infection, but showed a decreasing tendency which was inverse to the increase in viral titers (Figure 3A). It then began to decline at 4 dpi when the anamnestic IgG response was revived (Figure 2A), falling quickly into the negative detection range in spite of a rebound <3-fold at 12 dpi without a surge of viral burden (Figure 1). Moreover, the *TLR-7* response in the blood did not correlated directly with significantly better control of virus replication (*P* > 0.05) (Figure 4). *MDA5* was slightly upregulated only at 0.5 dpi, while *TLR-3* and *RIG-1* were downregulated for the duration of the experiment. These data indicated that *TLR-7* plays a vital role in initiating the innate response to DHAV-1 infection in mature ducks.

### IL and IFN Gene Expression Was Upregulated during DHAV-1 Infection in Mature Ducks

Interleukins (*IL-1β*/*2*/*4*/*6*) (Figure 3B) and IFNs (*IFN-α/β/γ*) (Figure 3C) were induced with characteristic expression patterns. *IL-4* was the most strongly elevated among these immune-related genes, which was observed as a sustained increase for the duration of the experiment regardless of the variation in viremia. *IL-1β* exhibited the lowest expression levels among the ILs tested, being downregulated nearly the entire time and upregulated briefly at 0.5 and 28 dpi. Contrary to *IL-1β*, the expression of *IL-2* was highly upregulated for most of the time between days 2 and 28, although with a transient decline at 4 dpi. In addition, *IL-6* was also upregulated for most of the time with various expression forms.

Resembling the expression of *IL-1β, IFN-α* expression was downregulated over the duration of the experiment except for a fivefold upregulation solely at 0.5 dpi. *IFN-β* increased coincidentally with the replication of virus and reached a peak level at 2 dpi. From 4 dpi on, the upregulation of *IFN-β* remained steady with an exception at 6 dpi. Different from the prompt upregulation of type I IFN genes, an increase in *IFN-γ* mRNA levels was observed first at day 6, nearly 1 week after the onset of viremia. Although it was upregulated continuously from 10 dpi accompanied by a decline in virus titer, *IFN-γ* expression did not correlate directly with viral clearance (*P* > 0.05). Notably, among these immune-related genes, only *IL-6* was correlated directly with spontaneous clearance of the virus (*P* < 0.05) (Figure 4).

### *MHC*s but Not *BAFF* Gene Expression Was Elevated in Mature Ducks Exposed to DHAV-1 Infection

To confirm whether MHC-I and MHC-II molecules were involved in the host immune responses to DHAV-1, we examined their gene expression within 1 month after infection (Figure 3D). The expression of *MHC-I* was promptly highly upregulated at 0.5 dpi. Meanwhile, *MHC-II* was highly upregulated from 0.5 to 10 dpi except for a sudden downregulation at 6 dpi. Although the expression of both genes was highly elevated at the onset of viremia, only *MHC-II* levels were correlated with the virus titer (*P* < 0.05) (Figure 4). Moreover, the expression level of *MHC-II* was higher than that of *MHC-I* overall. In addition, *BAFF* showed a distinctly unique pattern without any upregulation for the duration of the experiment.

### Expression Patterns of *β-Defensin* and Chemokines in DHAV-Infected Mature Ducks

Parallel to *IL-4*, expression of *β-defensin* showed a constant increase from the onset of viremia (Figure 3E) and did not correlate directly with spontaneous clearance of the virus (Figure 4). Although *CCL-19* failed to respond to the virus for the duration of the experiment, the expression of *CCL-21* was upregulated from 8 to 12 dpi (Figure 3E).

### Interaction between the Virus and the Immune System

To understand the impact of the virus on immune networks, and the interaction between the innate immune system and the humoral immune response, the Pearson correlation analysis was adopted (Figure 4). In total, 53 pairs of factors were highly correlated (*P* < 0.05 at least). Based on the analysis, coadjustments between the innate and adaptive immune systems were confirmed. Although *TLR-3* was downregulated over the duration of the experiment, *TLR-3* and *TLR-7* were the two PRR genes whose expression was most correlated with that of other cytokines. Moreover, three subtypes of antibody were also correlated positively or negatively with certain innate cytokines. Notably, the only factors that were involved in viral clearance directly were *IL-6* and IgG antibody.

## Discussion

Duck hepatitis A virus 1 infection in mature ducks has been proposed as an attractive small-animal model for research of human hepatitis A virus (HAV) (8). This study provides critical detailed virological and immunological information that clarifies the outcome of DHAV-1 infection in mature ducks. Our studies on the clinical signs have shown that adult ducks infected with DHAV-1 are asymptomatic, although with an elevated serum ALT but a normal bilirubin (8). This undramatic infection pattern with a lack of visible evidence of disease seems to be one aspect of the “host–parasite” relationship between adult ducks and the hepatitis virus. It offers an advantage for dissecting the host response in asymptomatic hepatitis.

An important observation is that the ducks infected with DHAV-1 experienced acute as well as persistent phases of viremia, providing an opportunity to research the contributors to the distinctive outcome of DHAV-1 infection, and indicating that this model can also be used to study the underlying mechanism of chronic infection, which will extend the current studies on chronic hepatitis (30, 31). Studies involving hepatitis B virus (HBV) and hepatitis C virus (HCV) have showed that their RNAs cannot be detected until weeks later, which is usually associated with a latent phase of infection (32, 33). Our study showed that DHAV-1 RNA could be detected and reached the maximal level in the plasma within days after exposure, similar to the HAV infection in which the viral RNA was able to be detected at the early phase of infection without a latent phase (34). Notably, the maximum viral load detected during the studies of replication kinetics was lower than the inoculated amount, which was due to the alert innate immune response and preexisting IgG. Another intriguing finding of DHAV-1 kinetics was that virus levels significantly rebounded at 56 dpi, which was associated with virus re-propagation in the liver due to the generation of a large number of fibrocytes in which DHAV-1 was propagated massively at that time (8). Accompanying this rebound, levels of antibodies were increased again. Thus, the exact roles of this phenomenon in viral persistence or clearance needs further investigations.

The humoral immune response has been certified to clear hepatitis G virus spontaneously from the host (35), and a delayed humoral immune response has been reported to be associated with the persistence of HCV (36). The viremia of HAV was also terminate about the time that humoral immunity was detected (37). Detection of the various antibody classes proved that specific IgG, IgM, and IgA1 were all generated timely. Because specific antibody classes mediate different immune effector functions, a diverse repertoire of antibody classes is probably necessary to control viral infection (38, 39). Pearson correlation analysis suggested different relationships between specific antibodies and DHAV-1 in our study. Although the ducks had been vaccinated previously, the magnitude of the antibody response would not be enhanced in the experimental DHAV-1 infection. It has been previously reported that antibody responses to trinitrophenol and H5N1-inavtivated vaccine, while robust, were no greater evidently than the initial response (40, 41). The general nature of the duck antibody response ensures an actual interaction between the ducks and the virus. The antibody response of ducks was activated soon after infection. It was reported that the neutralization antibodies at a protective titer could be detected at 5 dpi (5). As expected, IgG was produced quickly and abundantly at 4 dpi as the predominant antibody in response to DHAV-1 in terms of the magnitude and longitude. Coinciding with the increase in the level of IgG, the viral burden began to decrease. Note that a second peak of IgG was observed at 84 dpi, which was caused by the recurrent viral replication at 56 dpi. The difference in magnitude of the two peaks was not significant (*P* > 0.05), validating the nature of the duck secondary antibody response. Whatever, the timely production of a robust IgG response against DHAV-1 infection, suggesting the ability of host responses to outpace virus propagation, ensures the ultimate clearance of DHAV-1 viremia.

Widely known, the existence of a specific IgM antibody is a mainstay marker of recent or ongoing infection (38, 42). The presence of any detectable antibody plus the absence of high-titer, IgM-specific antibodies is used to differentiate between past and current infection. A level of specific IgM lower than the cutoff point at 0 dpi accompanied by a positive IgG anti-DHAV-1 showed that the ducks selected had been vaccinated or exposed previously. Such a case is consistent with the vaccination of ducklings at 1-day-old and the absence of an outbreak of DHAV-1 during farm feeding. IgM is usually considered the product of a primary immune response and is undetectable after revaccination (43). Our finding of a quick rise in IgM anti-DHAV-1 levels to a peak was expected in this asymptomatic infection, implying that IgM antibodies will be produced regardless of the disease severity and that IgM response follows clinical DHAV-1 infection independent of prior immunization status. Traditionally, IgM is observed quickly following exposure to antigens and soon disappears. However, in this study, the IgM antibody persisted for a very prolonged period of time, as long as 224 days. The apparent persistence of IgM-specific HAV antibody in a number of patients after the disappearance of all clinical evidence of hepatitis has been well documented (44, 45). However, the intriguing unanswered question is whether the persistence of IgM-specific antibody correlates with the persistence of antigen stimulation in a clinically dormant state. Studies have shown that the persistence of IgM anti-HAV is not due to high titers of hepatitis A IgG antibody nor to rheumatoid factors (45, 46). Moreover, the percentage of patients in which ALT was normalized before or by disappearance of IgM anti-HAV was 72 and 15%, respectively (45). Similarly, our study showed that there was no direct correlation between the levels of IgG anti-DHAV-1 or ALT and the persistence of positive IgM anti-DHAV-1. Notably, it was apparent that the duration of virus was related to the persistent positivity of IgM anti-DHAV-1 (*P* < 0.05). In chronic hepatitis B infection, IgM-class antibody continues to be produced albeit usually at undetectable levels, which may be associated with immune suppression by HBV (47). Despite the level of persistent IgM anti-DHAV-1 being significantly lower than that in the acute phase, IgM anti-DHAV-1 was detectable until the disappearance of the virus. These data show that IgM could be a reliable marker of recent DHAV-1 infection, and that the virus has no immune suppression effect on IgM response. In addition, it is more reliable to use the effective value of IgM rather than simply a positive or negative determination. Moreover, due to the persistence of IgM, the presence of IgM might not be enough to discriminate acute from chronic infection. Thus, the rapid increase in concentration is a reliable way to discriminate acute from persistent or ongoing infection.

In general, serum IgA levels tend to be higher than IgM levels but considerably lower than IgG levels, and more than 90% of serum IgA is in the form of IgA1 (39). IgA1 specific to DHAV-1 was induced at the lowest level with respect to the levels of specific IgG and IgM, although the correlation with DHAV-1 level was not significant. A similar situation for serum immunoglobulin levels in chronic HCV infection has been described (18). Moreover, in cases with mild liver disease in chronic infection, serum IgA values approximated the controls (18), while in acute infection, serum IgA1 was elevated significantly (*P* < 0.05) (17). Serum IgA1 specific to DHAV-1 was significantly elevated during the acute infection compared with the control (*P* < 0.0001), and there was a significant difference between the serum IgA1 levels in the acute phase and the persistent phase (*P* < 0.0001). Serum IgA level is a biomarker indicating advanced fibrosis and cirrhosis in chronic viral hepatitis and non-alcoholic fatty liver disease (48–50). However, there was no correlation between the level of serum IgA1 and the mild hepatic fibrosis that has been described previously (*P* > 0.05) (8). Thus, whether the detectable level of serum IgA1 after the disappearance of virus was caused by a hepatic disorder was unknown, because the liver acts as a physiological disposer of circulating immune factors (17).

As anticipated, innate immune responses were active against the DHAV-1 infection in mature ducks. However, the innate immune defense was relatively weaker during the infection of mature ducks than in young ducklings, based on the magnitude and breadth of upregulation responses of PRRs that initiate the innate immune responses (3). As known, TLR-7 is an essential PRR to recognize single-stranded RNA viruses (51), thereby resulting in the generation of pro-inflammatory cytokines and IFNs. The upregulation of TLR-7 by several picornaviruses had been reported (52, 53). Moreover, TLR-7 played a role in activating the NK cells in foot-and-mouth disease virus (FMDV) infection, which are cytotoxic against FMDV-infected cells *in vitro* (54). In this study, *TLR-7* was upregulated against the infection of DHAV-1, although the upregulation was mainly within 4 days after infection, as IgG levels were negatively correlated with the expression of *TLR-7*. Another upregulated PRR was *MDA5*, with a slight and transient elevation at 0.5 dpi. Clearly, the expression of *TLR-3* and *RIG-1*/*MDA5* in mature ducks was significantly distinct from the upregulations in ducklings against DHAV-1 infection. The results approve the previous viewpoint that the age of ducks can affect the expression of PRRs (3). Moreover, the expression of *TLR-3, RIG-1*, and *MDA5* showed tissue specificity, which were expressed in lymphoid organs mostly (7). TLR-3 and RIG-1/MDA5 are known to recognize double-stranded RNA (dsRNA) generated in the replication cycle of virus (11, 12, 55). However, most RNA viruses have evolved strategies to sequester dsRNA by diverse mechanisms to avoid activation of these antiviral pathways. NS1 protein encoded by influenza virus is known to bind to dsRNA and therefore inhibits various antiviral pathways (56). HAV also has evolved an ability to suppress the production of type I IFNs by disrupting the signaling pathways of TLR-3 and RIG-1/MDA5 (57, 58). But the in-depth mechanism constrained the expression of *TLR-3, RIG-1*, and *MDA5* by DHAV-1 or its products is still unknown. *IFN-α*, correlated with four *PRRs*, was expressed highly only at 0.5 dpi, while *IFN-β* was expressed for most of the time within 1 month after infection. Despite utilizing the same receptor, *IFN-α* and *IFN-β* have unique and distinguishable biological functions, with *IFN-β* being mainly responsible for promoting viral persistence by affecting T cell activity (59). A 1-week delay in *IFN-γ* expression was observed, which was usually associated with the absence of a vigorous CD8+ T cell response. Similarly, it was found that the function of CD8+ T cells improved slowly in HAV infection (60). The comparison of *IL-4* to *IFN-γ* showed a remarkable incline to Th2 immune response in blood, which is involved in regulating the humoral immune response. Constitutive expression of *IL-4* has delayed the clearance of respiratory syncytial virus and influenza virus in mice by delaying or suppressing the development of virus-specific cytotoxic T cells (61, 62). Moreover, in HCV infection in human, IL-4 was also suggested to play a role in the development of chronicity (63). Continuous and high-level expression of *IL-4* was observed in our study, indicating that it may play a role in delayed DHAV-1 clearance. A low level of *MHC-I* also indicated a weak CD8+ T cell response. The re-elevation of *IFN-β* following return to a normal level of *TLR-7* shows the existence of other induction pathways, e.g., a positive feedback of IFN-β to an IFN-β induction cascade with the virus as a cofactor (64). *IL-6* production induced by injury or infection is an important SOS signal that coordinates activities of liver cells, macrophages, and lymphocytes. Recently, *IL-6* has been identified as being associated with spontaneous clearance of HCV infection (65). The upregulation of *IL-6* in our study immediately following infection, combined with the inverse correlation with DHAV-1, confirmed that *IL-6* was associated with spontaneous resolution of DHAV-1 infection. β-Defensin, a broad-spectrum antimicrobial peptide, has been shown to inhibit the replication of duck hepatitis virus *in vitro* (66). *In vivo, β-defensin* was indeed upregulated; however, there was no correlation between DHAV-1 titer and *β-defensin*. But, β-defensin and IL-2 cooperate to suppress the production of IgM and IgA1, and *vice versa*. This is because IL-2 can be bound to regulatory T cells expressing with IL-2 receptor and coexpressing CCR7, which is able to be recruited by the elevation of CCL-21 and therefore signal the suppressor function (67–69). Collectively, these observations suggest that the cytokine networks are pleiotropic and crucial in the outcome of DHAV-1 infection.

In conclusion, this study documented the replication kinetics of DHAV-1, DHAV-specific antibody kinetics, and DHAV-associated innate immune-related gene expression. Asymptomatic DHAV-1 infection in mature ducks produced acute as well as persistent hepatitis phases. Specific IgG antibody and *IL-6* in blood promoted the spontaneous clearance of DHAV-1 infection. Several possible factors associated with persistent infection were evident, which will be validated in a future study. The current experiment confirmed that it is feasible to mimic the virological and immunological aspects of human viral hepatitis using mature ducks infected with DHAV-1. These data extend the information on model hepatitis and provide the basis for future studies focused on defining the nature of mechanisms involved in the clearance and persistence of human hepatitis virus.

## Ethics Statement

This study was carried out in accordance with the recommendations of the ARRIVE guidelines (http://www.nc3rs.org.uk/arrive-guidelines). These experiments had been approved by the committee of experiment operational guidelines and animal welfare of Sichuan Agricultural University, China (approved permit number XF2014-18). All ducks were handled in full compliance with the animal welfare regulations and maintained according to the standard protocols.

## Author Contributions

SM, XO, AC, and MW designed the experiments; AC and MW contributed materials and experimental platforms; SM, XO, and DS acquired data; SM, XO, DS, ML, QY, YW, KS, XC, and AC analyzed and interpreted data; SM and XO wrote the manuscript; SM, XO, DZ, SC, RJ, XZ, and ML proofread the draft; all authors approved the final version of the manuscript.

## Conflict of Interest Statement

The authors declare that the research was conducted in the absence of any commercial or financial relationships that could be construed as a potential conflict of interest.
