# Supplementary material for: Virologic and Immunologic Characteristics in Mature Ducks with Acute Duck Hepatitis A Virus 1 Infection
Source: Front Immunol. 2017 Nov 16;8:1574. doi: 10.3389/fimmu.2017.01574 (PMC5696325; doi:10.3389/fimmu.2017.01574)
Supplement: Supplementary file 1 [file Table_1.PDF]

Table S1

Primer sequences used in gene expression profiles.

Gene	Forward primer	Reverse primer	Accession number	Reference
GAPDH	5’-ATGTTCzGTGATGGGTGTGAA-3’	5’-CTGTCTTCGTGTGTGGCTGT-3’	AY436595	[25]
IL-1β	5’-TCGACATCAACCAGAAGTGC-3’	5’-GAGCTTGTAGCCCTTGATGC-3’	DQ393268	[25]
IL-2	5’-GCCAAGAGCTGACCAACTTC-3’	5’-ATCGCCCACACTAAGAGCAT-3’	AF294323	[25]
IL-4	5’-CCTCCACGGTTGTTTTCGAG-3’	5’-GTTGGAGGGTTCTGTGGAGG-3’	XM_005024359.1	[24]
IL-6	5’-TTCGACGAGGAGAAATGCTT-3’	5’-CCTTATCGTCGTTGCCAGAT-3’	AB191038	[25]
IFN-α	5’-TCCTCCAACACCTCTTCGAC-3’	5’-GGGCTGTAGGTGTGGTTCTG-3’	EF053034	[25]
IFN-β	5’-CCTCAACCAGATCCAGCATT-3’	5’- GGATGAGGCTGTGAGAGGAG-3’	AY831397	[25]
IFN-γ	5’-GCTGATGGCAATCCTGTTTT-3’	5’- GGATTTTCAAGCCAGTCAGC-3’	AJ012254	[25]
MHC-I	5’-GAAGGAAGAGACTTCATTGCCTTGG-3’	5’-CTCTCCTCTCCAGTACGTCCTTCC-3’	AB115246	[25]
MHC-II	5’-CCACCTTTACCAGCTTCGAG-3’	5’-CCGTTCTTCATCCAGGTGAT-3’	AY905539	[25]
CCL-19	5’-CCAGGAAGGTCCCAAATAAA-3’	5’-GTAGTAGGAGGTGGAAGCAAGTC-3’	DR766004	[24]
CCL-21	5’-GGAGAAGCAGAAGAACCCCC-3’	5’-GGGAAAGCATCCGTCCTCTC-3’	DR764376	[24]
BAFF	5’-TGTGCACGTCATCCAACAGA-3’	5’-GCCACAGGAATGTGACAGGA-3’	DQ445092	[24]
β-defensin	5’-CCAGGTTTCTCCAGGATTGT-3’	5’-AACCCAAAGCAACTTCCAAC-3’	AY641439	[24]
TLR-3	5’-AACACTCCGCCTAAGTATCAT-3’	5’-CTATCCTCCACCCTTCAAAA-3’	JN573268	[24]
TLR-7	5’-CCTTTCCCAGAGAGCATTCA-3’	5’-TCAAGAAATATCAAGATAATCACATCA-3’	AY940195	[25]
RIG-1	5’-GCGTACCGCTATAACCCACA-3’	5’-CCTTGCTGGTTTTGAACGC-3’	AB772012.1	[24]
MDA-5	5’-GCTGAAGAAGGCCTGGACAT-3’	5’-TCCTCTGGACACGCTGAATG-3’	KJ451070.1	[24]
